# Machine learning constructs a T cell-related signature for predicting prognosis and drug sensitivity in ovarian cancer

**DOI:** 10.18632/aging.205536

**Published:** 2024-02-09

**Authors:** Yunzheng Zhang, Lipeng Pei

**Affiliations:** 1 Department of Obstetrics and Gynecology, General Hospital of Northern Theater Command, Shenyang 110015, China

**Keywords:** ovarian cancer, T cell, bioinformatics, immunotherapy

## Abstract

Background: The leading cause of death related to gynecologic cancer is ovarian cancer, which typically has a poor prognosis. T cells are referred to as key mediators of immunosurveillance and tumor eradication, and unbalanced regulation or lack of T cells in tumors result in immunotherapy resistance.

Methods: The identification of T cell related markers depended on single-cell RNA-seq analysis. Using data from multiple datasets, including TCGA, GSE14764, GSE26193, GSE26712, and GSE140082, we constructed a prognostic signature called TRS (T cell-related signature) using 10 different machine learning algorithms. The correlation between TRS and drug sensitivity were analyzed using the data from GSE91061 and IMvigor210 dataset.

Results: PlsRcox method based TRS was as a risk factor for the clinical outcome of ovarian cancer patients. In comparison with stage, grade and many prognostic signatures, the performance of our TRS in evaluating the clinical outcome was better in ovarian cancer. TRS-based risk score showed distinct association with the level of ESTIMATE score, immune-related function score and immune cells. Moreover, TRS could be used to predict the immunotherapy response and chemotherapy response in ovarian cancer.

Conclusion: In conclusion, we constructed a powerful TRS in ovarian cancer, which could accurately predict the clinical outcome of patients and be used to predict the immunotherapy response and chemotherapy response.

## INTRODUCTION

The leading cause of death related to gynecologic cancer is ovarian cancer, which typically has a poor prognosis [1]. Over 300,000 new cases are initially diagnosed with ovarian cancer globally by 2020, ranking 3.6% of all cancer diagnoses [2]. Lacking clinical symptoms and effective screening approaches in early stages, over half of patients are diagnosed with ovarian cancer at advanced diseases [3]. Multidisciplinary therapies have been for the managements of ovarian cancer patients. Chemoresistance and relapse of ovarian cancer are the primary reason for the management failure in ovarian cancer [4, 5]. These data suggest the vital role of identifying novel biomarkers evaluating the clinical outcome and therapy benefits of ovarian cancer.

The interaction between malignancies and immune microenvironment exerts key roles in the advancement, spread and therapy resistance of ovarian cancer [6, 7]. Immune T cell infiltration, excluded T cell infiltration and desert T cell infiltration patterns, could lead to different therapeutic effect [8]. T cells are referred to as vital mediators of immunosurveillance and cancer eradication. Unbalanced regulation or lack of T cells in tumors resulted in immunotherapy resistance [9]. CD8^+^ T cell related signature could predict the clinical outcome of renal cell carcinoma patients [10]. Moreover, in breast cancer, CD8^+^ T-cell model was correlated with clinical outcome and drug sensitivity [11]. Another study also showed that CD8^+^ T-cell based model could predict clinical outcome and therapy benefits in lung adenocarcinoma [12]. In head and neck squamous cell carcinoma, CD8^+^ T-cell model could accurately predict patients’ prognosis and serve as an index for clinical treatment [13]. Thus, elucidating the T cell-related markers expression pattern and constructing a signature may help us manage the clinical outcome in ovarian cancer.

Our investigation identified T cell related markers in ovarian cancer by single cell analysis. Moreover, we constructed a machine learning algorithm-based T cells-related signature (TRS) using one TCGA dataset (training cohort) and four GEO datasets (testing cohort). Our result may offer more evidence regarding the vital roles of T cells in the clinical outcome and drug sensitivity in ovarian cancer.

## MATERIALS AND METHODS

### Datasets

**Figure 1:**
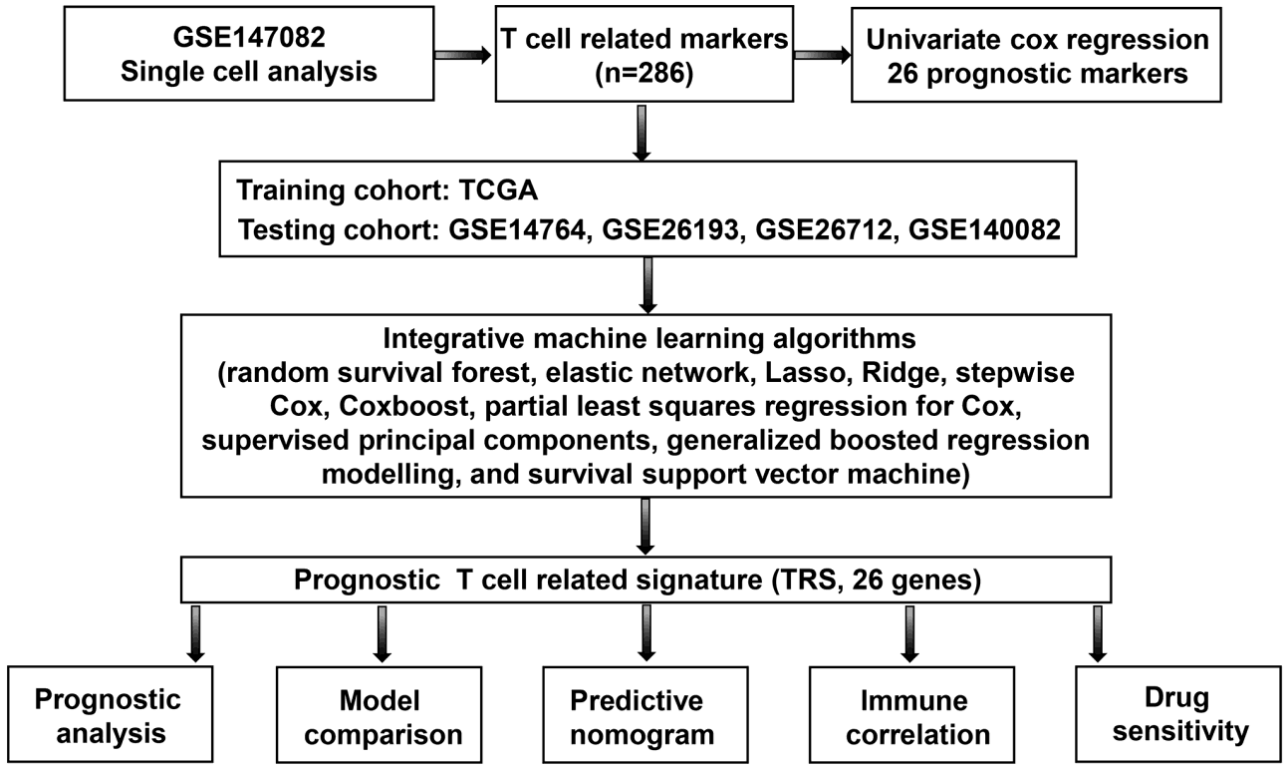
Workflow of the current study.

Work flow was shown in Figure 1. The single cell expression data of ovarian cancer was obtained from GSE147082 (*n* = 6) dataset. Ovarian cancer related bulk transcriptomics data (FKPM) were downloaded from TCGA (*n* = 374) database. GSE14764 (Platforms: GPL96, *n* = 80), GSE26193 (Platforms: GPL570, *n* = 107), GSE26712 (Platforms: GPL96, *n* = 185) and GSE140082 (Platforms: GPL14951, *n* = 380) datasets were used for verifying the TRS. Inclusion criteria for selecting ovarian cancer cases should be: (1) diagnosed with ovarian cancer using histological method; (2) complete data about overall survival. While metastatic ovarian cancer and ovarian cancer along with other types of cancer should be excluded in the study. IMvigor210 and GSE91061 were used as validation cohort for predicting immunotherapy benefit.

### Single-cell RNA-seq analysis

The procession of single cell expression dataset GSE147082 relied on “Seurat” R package [14]. Those genes that detected in less than 3 cells and cells with less than 50 detected gene numbers were ruled out. The mitochondria proportion was set as 5%. In order to normalize the expression data, we then performed principal component analysis. We then conducted unsupervised clustering analysis with UMAP methods [15]. In order to generate the marker genes of each cluster, we utilized “FindAllMarkers” function of “Seurat” R package and the threshold of the minimum cell population fraction in either of the two populations was set as 0.25. In order to identify the cell type of each cluster, we performed cell annotation analysis with “SingleR” package [16].

### Integrative machine learning algorithms constructed a prognostic T cells-related signature

The screening of the prognostic markers in ovarian cancer in TCGA dataset relied on univariate cox regression analysis (*p* < 0.05). The development of prognostic TRS relied on 10 integrative machine learning algorithms in ovarian cancer. The model with the highest average C-index in all datasets was suggested as the best model. Previous investigations have used similar machine learning algorithms [17–21]. After determining the optimal prognostic TRS, we could obtain the genes in the optimal prognostic TRS and the coefficient. On the basis of gene expressions and coefficient, we could calculate risk score of each ovarian cancer patients (risk score = the sum of the coefficient × gene expression).

### Evaluation of the performance of TRS

The generation of overall survival curve relied on “survival” package. Time ROC analysis was conducted to assess the performance of TRS in evaluating the prognosis of ovarian cancer with “timeROC” package. The C-indexes of TRS and stage and grade were calculated with “rms” package. Moreover, we also collected 25 ovarian cancer-related mRNA and lncRNA-related models randomly, and calculated their C-indexes. Considering clinical characters and TRS, univariate and multivariate cox analysis was conducted to identify the risk factors for the clinical outcome of ovarian cancer. We then constructed a predicting nomogram via “nomogramEx” R package on the basis of TRS and clinical characters in ovarian cancer.

### Correlation between risk score and immune microenvironment and genetic mutation

The Immune score and ESTIMATE score of each ovarian cancer patient were determined by ESTIMATE algorithm [22]. A total of 7 immune algorithms, including CIBERSORT were used to evaluate the relative proportions of infiltrating immune cells in ovarian cancer. The abundance of immune cells and the score of immune-related activities or functions were relied on single sample gene set enrichment analysis using “GSVA” package. The genetic landscape was drawn with “maftools” package. Moreover, GSEA was performed to identify the biological functions linked to KEGG pathways TRS based high and low-risk groups.

### Evaluation of the performance of TRS in predicting the drug sensitivity

From The Cancer Immunome Atlas (https://tcia.at/home), we generated the immunophenoscore (IPS) of ovarian cancer cases. The TIDE score of ovarian cancer cases were evaluated by TIDE methods. Drug sensitivity data were downloaded from Genomics of Drug Sensitivity in Cancer, with which we determined the half maximal inhibitory concentration (IC50) value in each ovarian cancer case using “oncoPredict” package.

### Availability of data and materials

The analyzed data sets generated during the study were sourced from the TCGA database (https://portal.gdc.cancer.gov/repository) and GEO database (https://www.ncbi.nlm.nih.gov/geo).

## RESULTS

### Single-cell analysis revealed cell subtypes and T cell related markers

**Figure 2:**
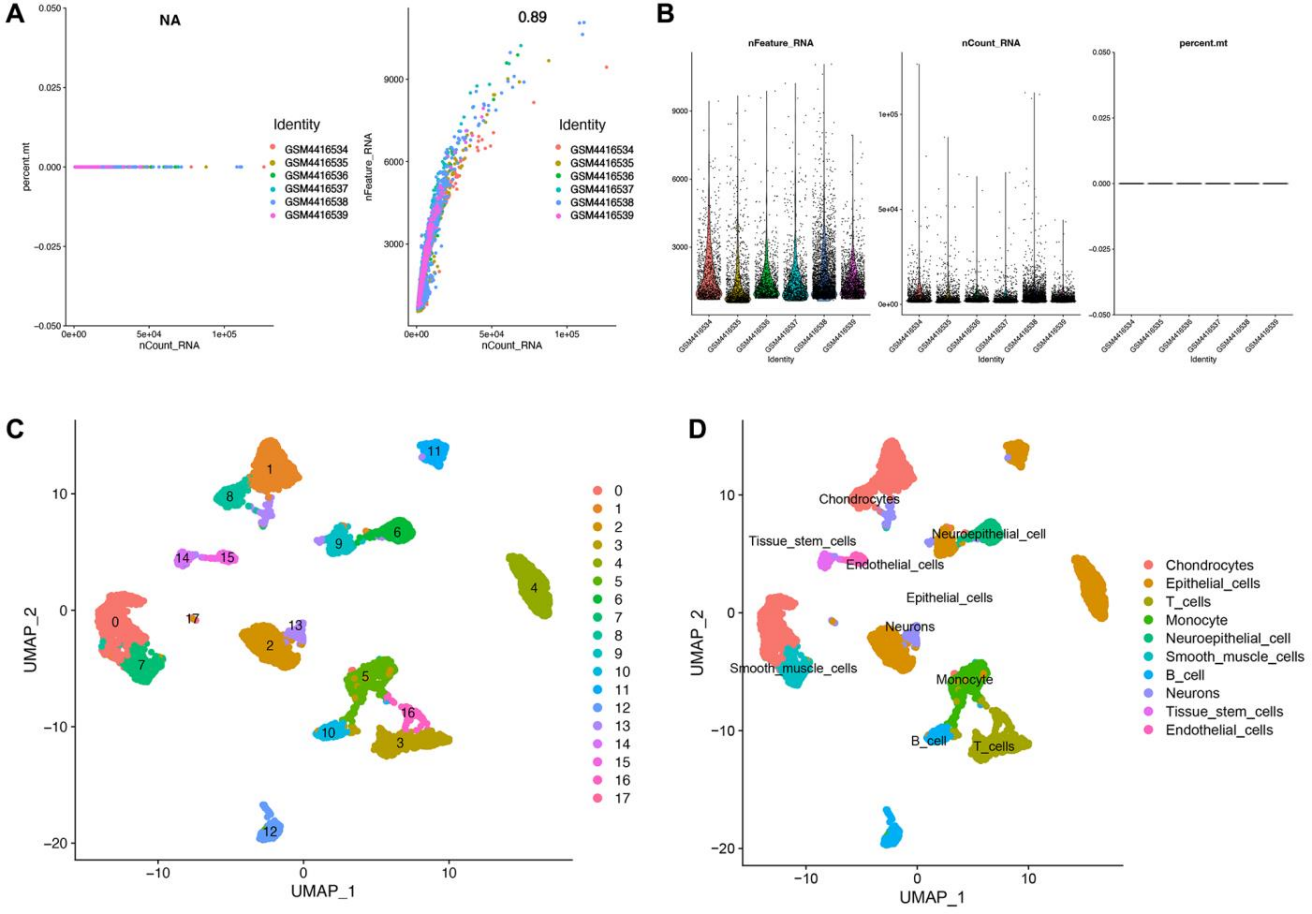
**Identification of T cell-related markers in ovarian cancer.** (**A**) Post quality control filtering of each sequenced cell. (**B**) Association analysis between nFeature and nCount. (**C**) A total of 17 clusters of all samples were identified after UMAP analysis. (**D**) A total of 10 subtypes of cells were identified based on SingleR annotation methods.

A strong positive correlation (Cor = 0.89) was obtained between the number of genes and the sequencing depth after sample normalization (Figure 2A). We obtained 9885 high-quality cell samples from 6 ovarian cancer tissues after stringent quality control metrics (Figure 2B). These cell samples could be clustered into 17 clusters based on UMAP analysis (Figure 2C). Cell annotating performed with SingleR technique identified 10 types of cells, including T cells, B cells, =, Endothelial cells, Neuroepithelial cells, and Neurons etc. (Figure 2D). And 286 T cell-related markers were obtained (Supplementary Table 1).

### Machine learning algorithms based prognostic TRS

**Figure 3:**
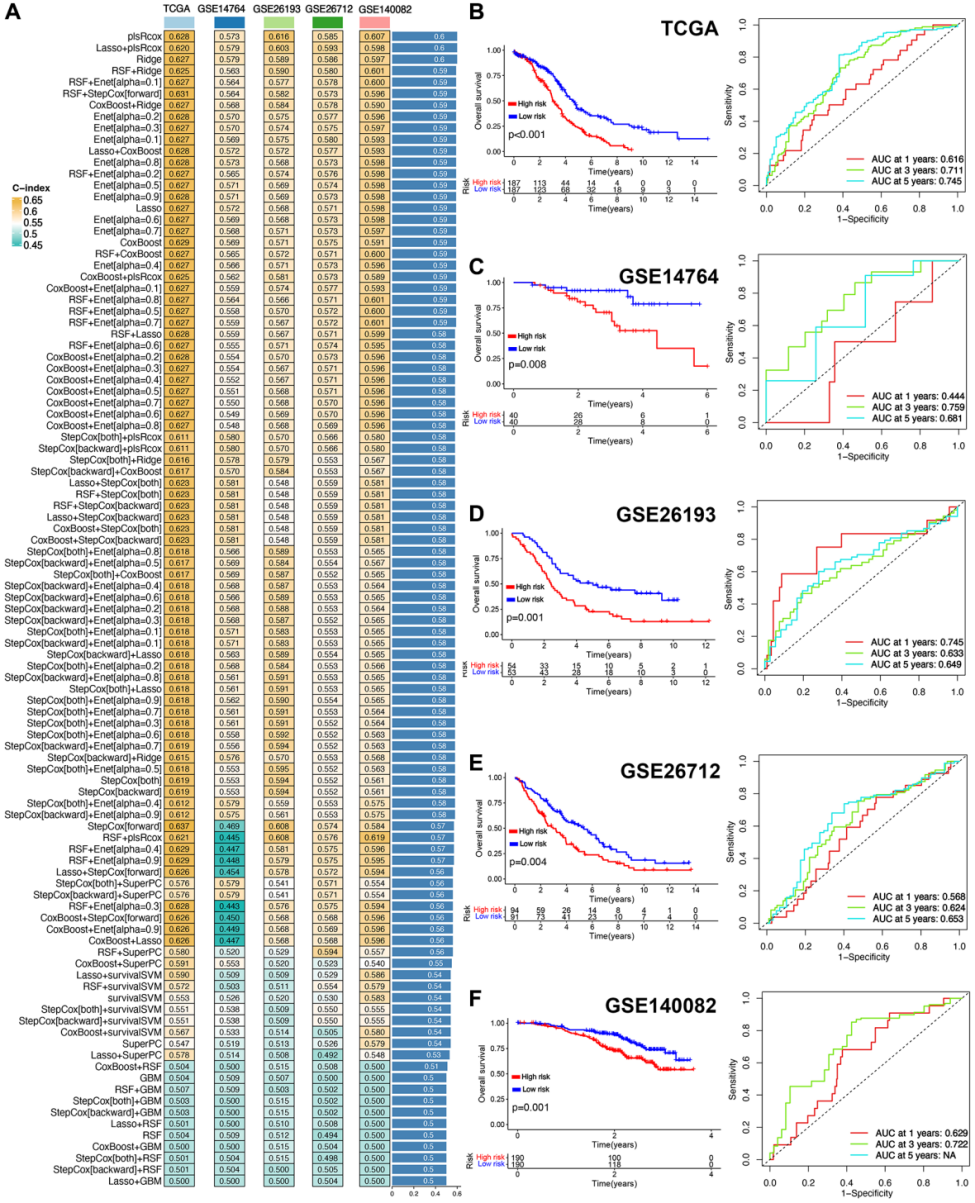
**Prognostic T cell-related signature (TRS) developed with integrative machine learning analysis.** (**A**) The C-index of each prognostic model constructed by 10 machine learning algorithms and 101 kinds of combinations in training and testing cohort. The survival curve and corresponding ROC curve of ovarian cancer with high and low-risk score in TCGA (**B**), GSE14764 (**C**), GSE26193 (**D**), GSE26172 (**E**) and GSE140082 (**F**) cohort.

Based on 286 T cell-related markers, we performed univariate cox analysis and identified 26 potential prognostic biomarkers (Supplementary Table 2). These 26 potential prognostic biomarkers were submitted into integrative procedure including 10 machine learning methods, which could conduct a prognostic TRS. Figure 3A showed the C-index of 101 kinds of prediction models in all datasets. The plsRcox method-based model was referred as the best model and it had a highest average C-index being 0.60 (Figure 3A). And a final set of 26 T cell-related genes were used to construct the TRS (Supplementary Table 2). Classification of high and low-risk groups relied on the medium value of risk score was the cutoff. As expected, in TCGA cohort, high-risk score indicated a poor OS rate in ovarian cancer with an AUCs of 1-, 3-, and 5-year ROC curve being 0.616, 0.711 and 0.745, respectively (Figure 3B, *p* < 0.001). In testing cohort, the data indicated a poor clinical outcome in high-risk score group in GSE14764, GSE26193, GSE26712 and GSE140082 cohort (Figure 3C–3F, all *p* < 0.05). ROC analysis measured the discrimination of TRS, with 1-, 3-, and 5-year AUCs of 0.444, 0.759, and 0.681 in GSE14764 dataset; 0.745, 0.633, and 0.649 in GSE26193 dataset; 0.568, 0.624, and 0.653 in GSE26712 dataset; 0.629, and 0.73 in GSE140082 dataset, respectively (Figure 3C–3F). The risk score, survival status, and gene expression of TRS of all cohorts could be seen in Supplementary Figure 1A–1E.

### Evaluation of the performance of TRS

**Figure 4:**
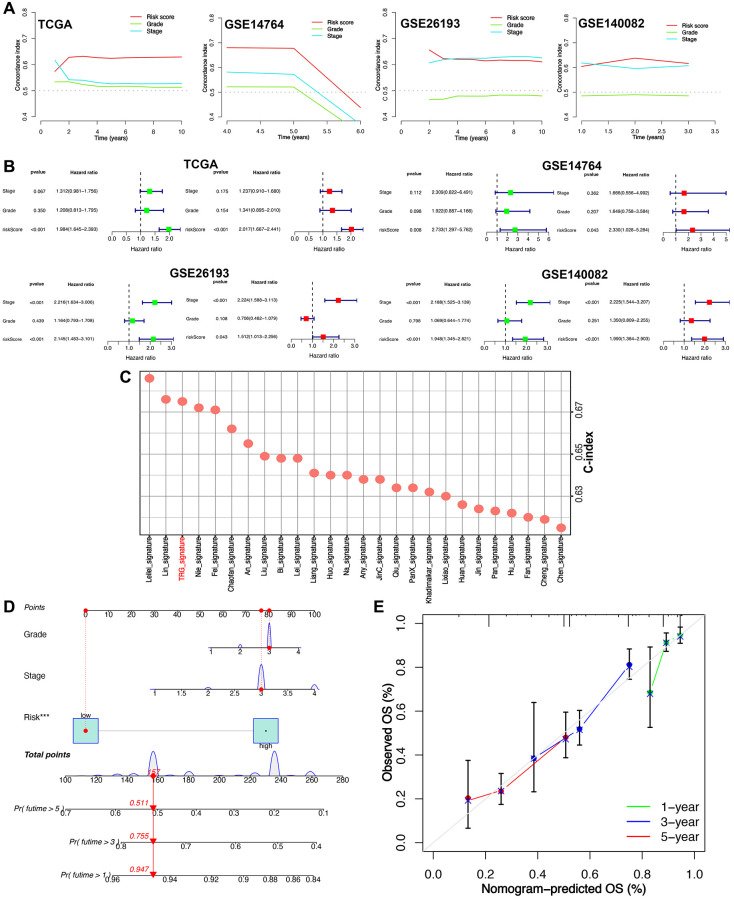
**The role of T cell-related signature (TRS) in predicting the prognosis of ovarian cancer.** (**A**) C-index evaluated the overall survival rate of ovarian cancer patients in training and testing cohort. (**B**) Univariate and multivariate cox regression analysis considering grade, stage and TRS in training and testing cohort. (**C**) C-index of TRS and other established signatures evaluated discrimination of TRS in predicting the prognosis of ovarian cancer patients. (**D**, **E**) Prediction nomogram for predicting the 1-, 3-, and 5-year OS rate of ovarian cancer.

As shown in Figure 4A, compared with grade and FIGO stage, risk score had a highest C-index in all cohort. However, there was no clinical information in GSE26712 cohort. Moreover, risk score was a risk factor for the clinical outcome of ovarian cancer in all cohorts (Figure 4B). Supplementary Table 3 showed the C-index of our TRS and 25 random prognostic models () were also calculated. And the data showed that the C- index most of these prognostic signatures was lower than the current TRS (Figure 4C). These results suggested that our TRS had a better performance in evaluating the prognosis of ovarian cancer cases. Based on TRS, stage and grade, we then developed a survival prediction nomogram (Figure 4D, 4E), with which the clinicians could evaluate the clinical outcome of ovarian cancer patients.

### TRS showed significant correlation with tumor microenvironment in ovarian cancer

**Figure 5:**
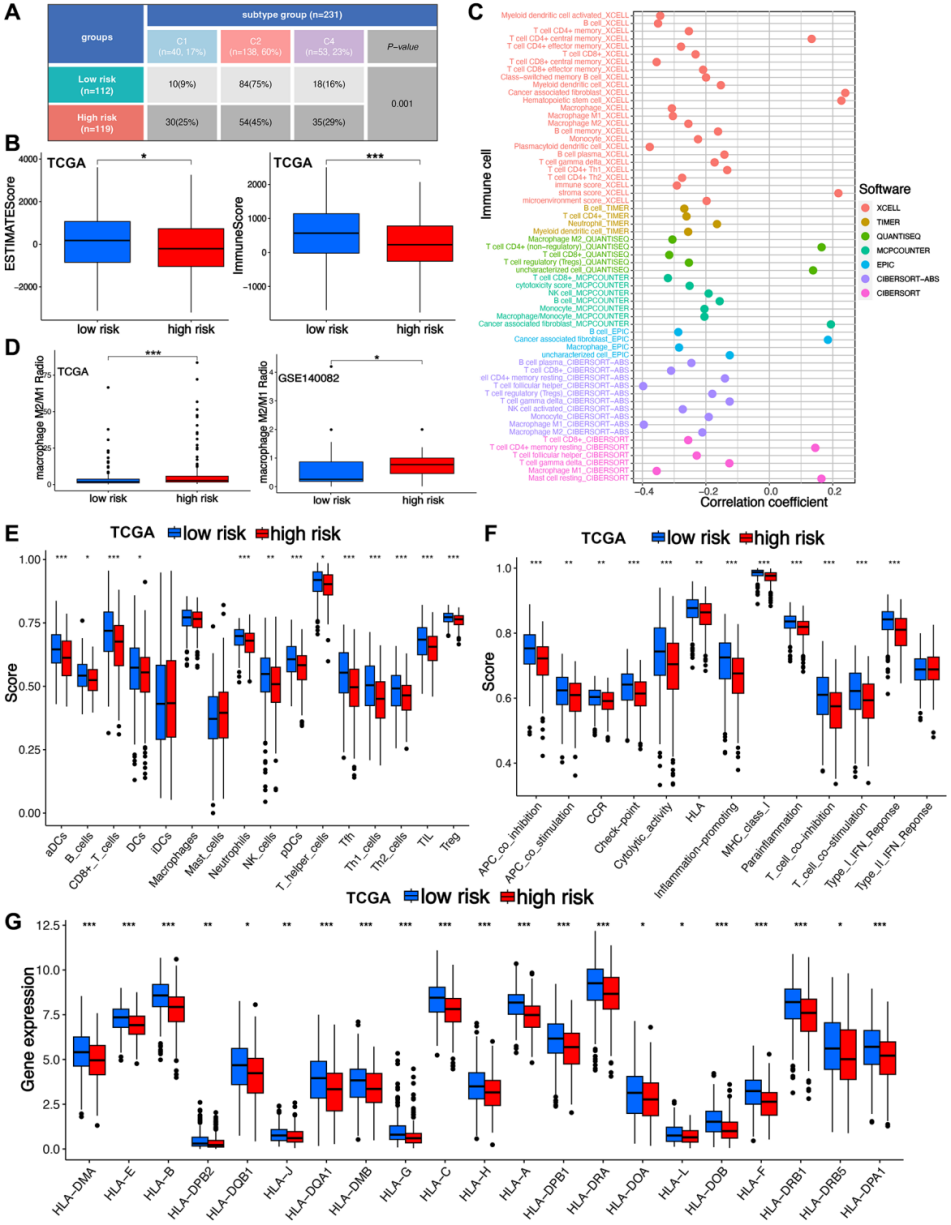
**Dissection of T cell-related signature (TRS)-based tumor microenvironment (TME).** (**A**) Tumor immune landscape in ovarian cancer with high and low-risk score. (**B**) The TME score difference in different risk score group of ovarian cancer. (**C**) The correlation between TRS and immune infiltration in ovarian cancer. (**D**) The level of macrophages M2/M1 proportion in ovarian cancer patients with high and low-risk score in TCGA and GSE140082 cohort. The difference of the score of immune cells (**E**), immune-related functions (**F**) and HLA-related genes (**G**) in different risk score group of ovarian cancer. ^*^p < 0.05, ^**^p < 0.01, ^***^p < 0.001.

Study had suggested six types of tumor immune landscape [22]. Most of TCGA ovarian cancer cases were IFN-g dominant(C2) type and lymphocyte depleted(C4) type ranked a higher proportion in high-risk score group compared with low-risk score group (Figure 5A, *p* = 0.001). Further analysis suggested a higher ESTIMATE score and Immunes core in low-risk score group (Figure 5B, all *p* < 0.05). Moreover, risk score had a negative correlation with the abundance of most of immune cells (Figure 5C). The result of the current study showed that low-risk score indicated a lower level of s M2/M1 proportion in TCGA and GSE140082 cohort (Figure 5D). Moreover, ovarian cancer patients with high-risk score correlated a lower level of T cells, B cells, NK cells, Th1 cells, Th2 cells and neutrophils (Figure 5E). Ovarian cancer cases with high-risk score demonstrated a lower score of immune related functions in ovarian cancer, including cytolytic activity and inflammation promoting (Figure 5F, all *p* < 0.05). Further analysis revealed that high-risk score indicated a lower level of most of HLA-related genes (Figure 5G).

### TRS-based treatment strategy for ovarian cancer

**Figure 6:**
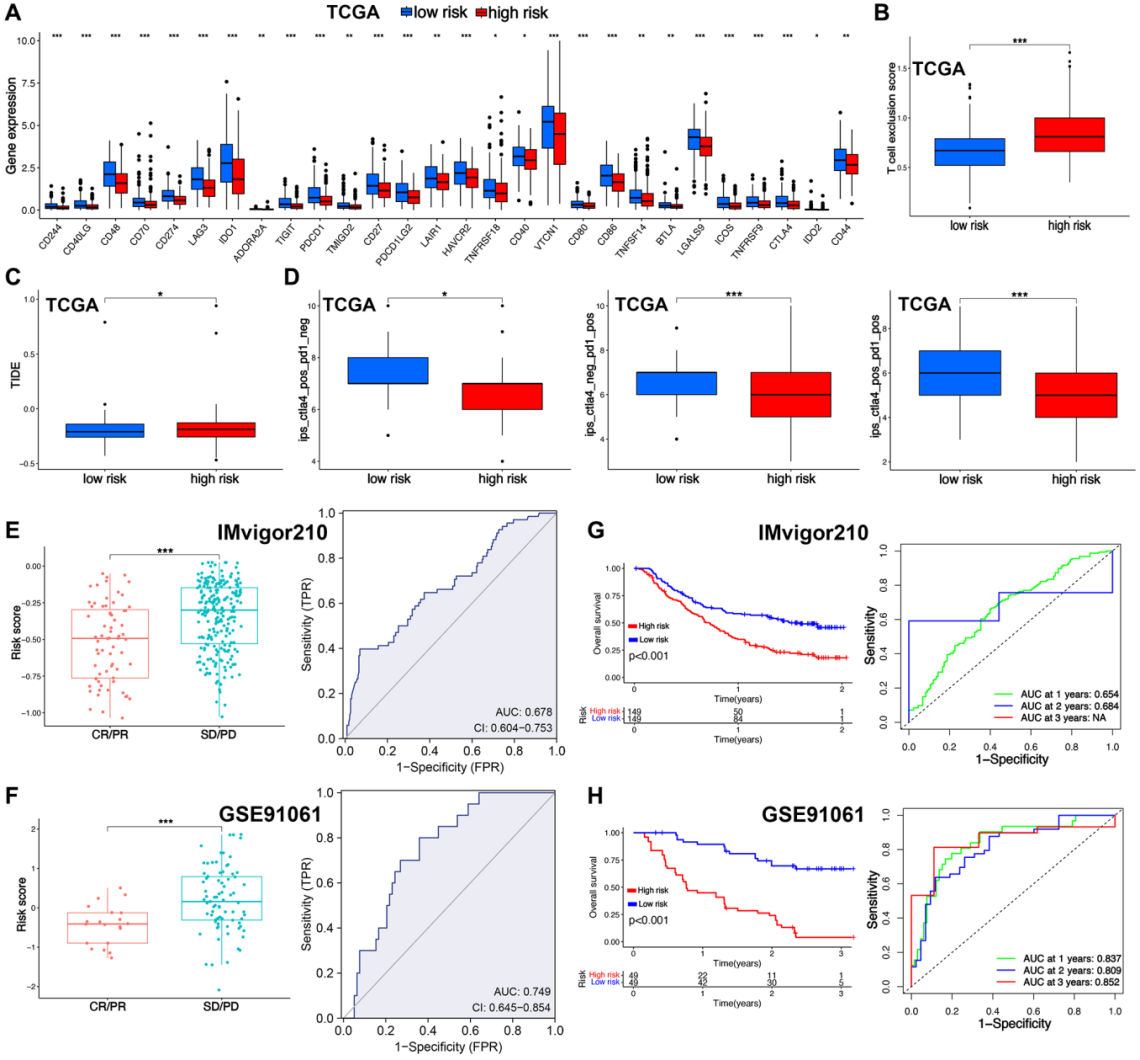
**T cell-related signature (TRS)-based treatment strategy for ovarian cancer.** The level of immune checkpoints (**A**), T cell exclusion score (**B**), TIDE score (**C**), immunophenoscore (**D**) in ovarian cancer patients with high and low-risk score. The risk score in CR/PR and SD/PD group and corresponding ROC curve in GSE91061 (**E**) and IMvigor210 (**F**) cohort. The OS curve in patients with high and low-risk score in GSE91061 (**G**) and IMvigor210 (**H**) cohort. ^*^p < 0.05, ^**^p < 0.01, ^***^p < 0.001.

**Figure 7:**
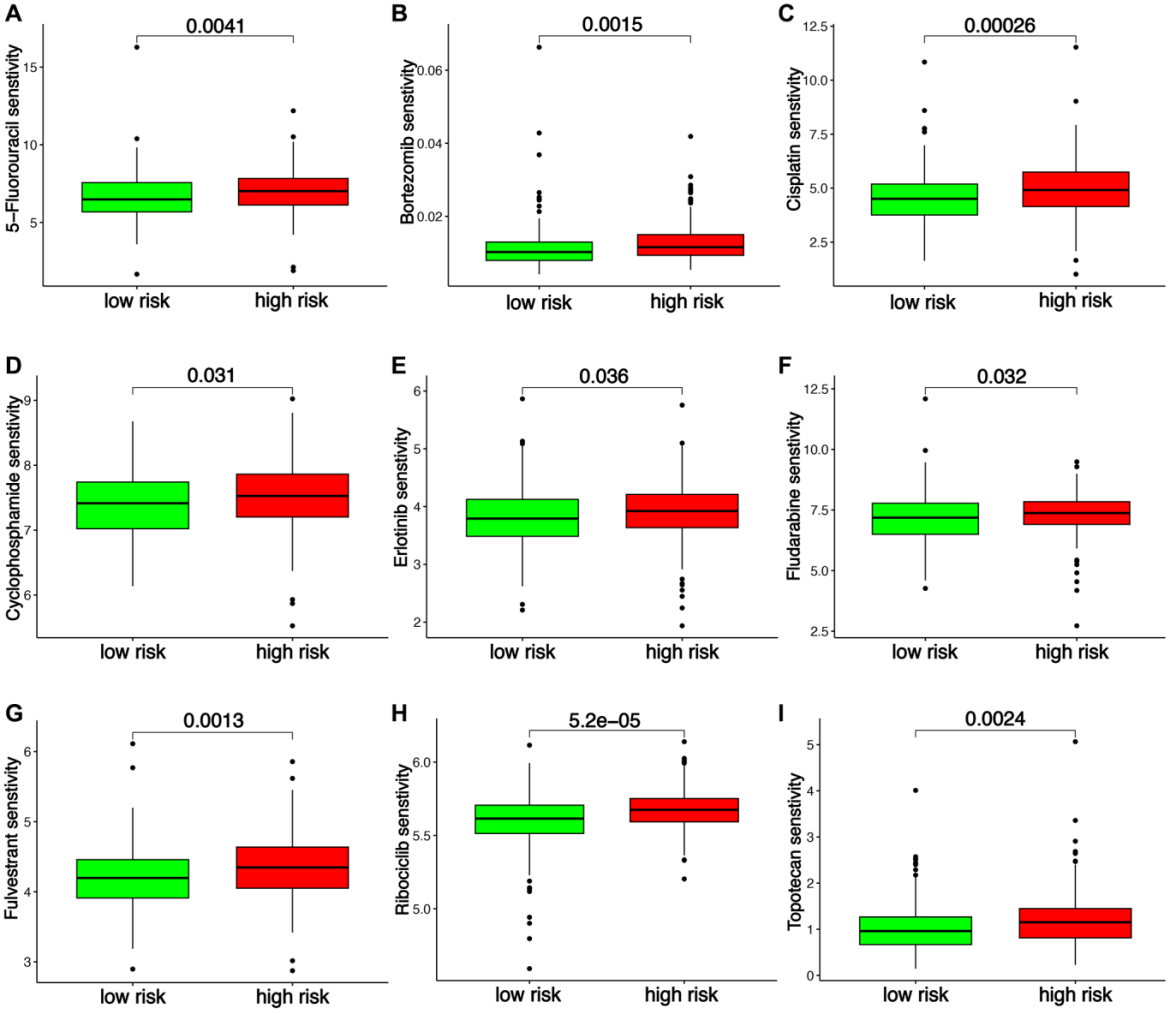
**T cell-related signature (TRS)-based treatment strategy for ovarian cancer.** The IC50 values of 5-Fluorouracil (**A**), Bortezomib (**B**), Cisplatin (**C**), Cyclophosphamide (**D**), Erlotinib (**E**), Fludarabine (**F**), Fulvestrant (**G**), Ribociclib (**H**) and Topotecan (**I**) in different risk score group of ovarian cancer.

Several indicators were then used to assess the functions of TRS in predicting immunotherapy benefits in ovarian cancer. As shown in Figure 6A, higher expression of many immune checkpoints was found in low-risk score group (all *p* < 0.05). TIDE score and T cell exclusion score could predict the immunotherapy benefits [23, 24]. Low TIDE score and T cell exclusion score indicated a better response to immunotherapy. As shown in Figure 6B, 6C, low-risk score group had a lower T cell exclusion score in ovarian cancer and TIDE score (all *p* < 0.05). IPS was an indicator for predicting the response to immunotherapy [25]. Low-risk score group had a increased IPS of anti-CTLA4 and anti-PD1 (Figure 6D, *p* < 0.05). In IMvigor210 cohort and GSE91061 cohorts, low-risk score was obtained in patients in CR/PR group with an AUC of 0.678 and 0.749 in ROC curve (Figure 6E, 6F, *p* < 0.001). Moreover, high-risk score indicated a poor OS rate in IMvigor210 dataset and GSE91061 dataset, with 1-, 3-, and 5-year AUCs of 0.654, 0.684, and NA; and 0.837, 0.809 and 0.852, respectively. (Figure 6G, 6H). Chemotherapy and endocrinotherapy were important therapeutic measures for ovarian cancer. We also detected the IC5O value of common drugs in high and low-risk score groups. As shown in Figure 7A–7I, the IC50 values of 5-Fluorouracil, Bortezomib, Cisplatin, Cyclophosphamide, Erlotinib, Fludarabine, Fulvestrant, Ribociclib, Topotecan were higher in high-risk score group versus low-risk score group (all *p* < 0.05), demonstrated that low-risk score had a better response to chemotherapy, endocrinotherapy, target therapy in ovarian cancer.

### TRS-based mutation landscape in ovarian cancer

The mutation landscape of ovarian cancer patients with low and high-risk scores were shown in Supplementary Figure 2A, 2B. TP53, TTN, and CSMD3 were top three most frequently mutated genes. A higher tumor mutational burden (TMB) score was seen in ovarian cancer patients with low-risk score (Supplementary Figure 2C, *p* = 0.0018). Moreover, negative correlation was seen between risk score and TMB score (Supplementary Figure 2D, *p* = 0.00055). As shown in Supplementary Figure 2E, 2F, low TMB score and high-risk score indicated a poor clinical outcome (*p* < 0.001).

### TRS-based functional enrichment difference in ovarian cancer

The data revealed that high-risk score was mainly linked to ECM receptor interaction, focal adhesion, melanoma, pathways in cancer and ribosome (Supplementary Figure 3A). Low-risk score was mainly linked to antigen processing and presentation and type I diabetes mellitus (Supplementary Figure 3B).

### TRS-based unsupervised clustering

Consensus clustering was conducted to identify unidentified subclasses in ovarian cancer. As shown in Supplementary Figure 4A, 4B, ovarian cancer patients could be well clustered into three subtypes according to the consensus CDF and delta area. Among these three clusters, cluster 1 had a best OS rate compared with cluster 2/3 in ovarian cancer (Supplementary Figure 4C, *p* < 0.001). Most of cluster 1 patients was correlated with low-risk while most of cluster 2 was correlated with high-risk (Supplementary Figure 4D). Further tSNE indicated significant differences of TRS gene expression among these three clusters (Supplementary Figure 4E). Moreover, these three clusters had a significant difference in TME. As shown in Supplementary Figure 4F, cluster 1 had a highest abundance of immune cells while cluster 2 had a lowest abundance. As we could see in Supplementary Figure 4G, 4H, ovarian cancer in cluster 1 had a highest level of ESTIMATE score, Immune score, and immune checkpoints (all *p* < 0.05).

## DISCUSSION

As one of the most common malignancies among women, ovarian cancer could result in poor prognosis [26]. Immune TME imbalance is one of the most conspicuous features of ovarian cancer [27]. The TME contains of immune cells stromal cells, and tumor cells [28]. Immune cells mediating the adaptive immune responses exerted a crucial function in tumor progression, thus affecting the prognosis of patients [12]. As one of predominant antitumor effector cells in the TME, T cell acted as a cytotoxic role and exerted vital roles in cancer cell clearance [29].

In our study, single cell analysis was performed to identify T cell related markers. After that, univariate cox analysis was conducted to screen out 26 novel prognostic markers for ovarian cancer. An integrative pipeline was developed to construct a powerful TRS using10 machine learning algorithms. Among 101 kinds of prognostic models, the plsRcox method-based model was referred as the best model and it had a highest average C-index being 0.60. Interestingly, TRS was as an independent risk factor for the clinical outcome in ovarian cancer. Prognosis analysis suggested a poor OS rate in high-risk score group. Moreover, the performance of TRS in predicting the clinical outcome of ovarian cancer cases was better than stage and grade.

In fact, many prognostic signatures have been developed in ovarian cancer. Immune-related signature could be used to evaluate the prognosis of ovarian cancer [30]. A panel of glycometabolism-related signature was linked to the prognosis for ovarian cancer [31]. Zhang et al. also constructed an oxidative stress-related signature predicting OS in ovarian cancer [32]. Moreover, glycolysis-based model [33], transcription factors related model [34], ferroptosis based model [35] and invasion-based model [36] could be used to evaluate the prognosis of ovarian cancer patients.

GSEA analysis indicated that high-risk score was mainly linked to focal adhesion, melanoma, pathways in cancer and ribosome. While patients with low-risk score were mainly correlated with antigen processing and presentation and type I diabetes mellitus. Thus, high-risk score was mainly correlated with pathways involved in tumor progression, which may be the reason why high-risk score group had a poor prognosis in ovarian cancer. While low-risk score ovarian cancer patients may be mainly correlated with pathways involved in immune response.

Our study also found that high-risk score group had a lower level of ESTIMATE score, Immune score, lower abundance of T cells, B cells, NK cells, Th1 cells, Th2 cells and neutrophils, lower level of most of HLA-related genes, and higher macrophages M2/M1 proportion. Higher Estimate scores indicate the lower the tumor purity [37]. T cells and NK cells play a vital role in eradicating tumor cells [38]. These explains why low-risk score group have a better clinical outcome. Studies highlighted the vital role of immunotherapy in the treatment of cancer. Many medications targeting PD-1, PD-L1 or CTLA4, such as nivolumab and pembrolizumab, could be used to manage many types of cancer in the first-line therapy [39, 40]. The current evidence showed that immunotherapy response rates among ovarian cancer patients remain modest [41]. Further study focused on immunotherapy response in ovarian cancer need to be performed. The current study used various indicators to assess the functions of TRS in predicting the immunotherapy benefits in ovarian cancer. The results revealed that ovarian cancer patients with high-risk score had a higher IPS, TMB score, and TME score and lower TIDE score. High TMB score indicated more neoantigens, resulting in that the tumor would be attacked by a large number of tumor-specific T cells [42]. Thus, TRS could be used to predict the immunotherapy response and chemotherapy response in ovarian cancer. In addition to surgery, chemotherapy was one of most key measures for treating ovarian cancer. Chemoresistance was refer as the major causes for the treating failure of ovarian cancer [43]. Thus, low-risk score indicated a better response to chemotherapy in ovarian cancer.

Some limitations could be found in our study. All the analyses were performed at RNA level, not representing the results of protein levels. Moreover, the level and prognosis of TRS in ovarian cancer should be verified with clinical tissues.

## CONCLUSION

In conclusion, we constructed a powerful TRS in ovarian cancer, which could accurately predict the clinical outcome of patients and be used to predict the immunotherapy response and chemotherapy response.

## Supplementary Materials

Supplementary Figures

Supplementary Table 1

Supplementary Tables 2 and 3

## References

[1] Armstrong DK, Alvarez RD, Bakkum-Gamez JN, Barroilhet L, Behbakht K, Berchuck A, Chen LM, Cristea M, DeRosa M, Eisenhauer EL, Gershenson DM, Gray HJ, Grisham R, et al. Ovarian Cancer, Version 2.2020, NCCN Clinical Practice Guidelines in Oncology. J Natl Compr Canc Netw. 2021; 19:191–226. 10.6004/jnccn.2021.0007 33545690

[2] Feng J, Yu Y, Yin W, Qian S. Development and verification of a 7-lncRNA prognostic model based on tumor immunity for patients with ovarian cancer. J Ovarian Res. 2023; 16:31. 10.1186/s13048-023-01099-0 36739404 PMC9898952

[3] Ye Y, Dai Q, Qi H. A novel defined pyroptosis-related gene signature for predicting the prognosis of ovarian cancer. Cell Death Discov. 2021; 7:71. 10.1038/s41420-021-00451-x 33828074 PMC8026591

[4] Jiang Y, Wang C, Zhou S. Targeting tumor microenvironment in ovarian cancer: Premise and promise. Biochim Biophys Acta Rev Cancer. 2020; 1873:188361. 10.1016/j.bbcan.2020.188361 32234508

[5] Kuroki L, Guntupalli SR. Treatment of epithelial ovarian cancer. BMJ. 2020; 371:m3773. 10.1136/bmj.m3773 33168565

[6] Hinshaw DC, Shevde LA. The Tumor Microenvironment Innately Modulates Cancer Progression. Cancer Res. 2019; 79:4557–66. 10.1158/0008-5472.CAN-18-3962 31350295 PMC6744958

[7] Dai Y, Qiang W, Lin K, Gui Y, Lan X, Wang D. An immune-related gene signature for predicting survival and immunotherapy efficacy in hepatocellular carcinoma. Cancer Immunol Immunother. 2021; 70:967–79. 10.1007/s00262-020-02743-0 33089373 PMC10992402

[8] Hornburg M, Desbois M, Lu S, Guan Y, Lo AA, Kaufman S, Elrod A, Lotstein A, DesRochers TM, Munoz-Rodriguez JL, Wang X, Giltnane J, Mayba O, et al. Single-cell dissection of cellular components and interactions shaping the tumor immune phenotypes in ovarian cancer. Cancer Cell. 2021; 39:928–44.e6. 10.1016/j.ccell.2021.04.004 33961783

[9] Shi X, Dong A, Jia X, Zheng G, Wang N, Wang Y, Yang C, Lu J, Yang Y. Integrated analysis of single-cell and bulk RNA-sequencing identifies a signature based on T-cell marker genes to predict prognosis and therapeutic response in lung squamous cell carcinoma. Front Immunol. 2022; 13:992990. 10.3389/fimmu.2022.992990 36311764 PMC9614104

[10] Wang J, Huang F, Zhao J, Huang P, Tan J, Huang M, Ma R, Xiao Y, He S, Wang Z, Shen J, Lu H, Meng L. Tumor-Infiltrated CD8+ T Cell 10-Gene Signature Related to Clear Cell Renal Cell Carcinoma Prognosis. Front Immunol. 2022; 13:930921. 10.3389/fimmu.2022.930921 35812454 PMC9263606

[11] Dong H, Xie C, Yao Z, Zhao R, Lin Y, Luo Y, Chen S, Qin Y, Chen Y, Zhang H. PTPRO-related CD8^+^ T-cell signatures predict prognosis and immunotherapy response in patients with breast cancer. Front Immunol. 2022; 13:947841. 10.3389/fimmu.2022.947841 36003382 PMC9393709

[12] Zhang M, Ma J, Guo Q, Ding S, Wang Y, Pu H. CD8^+^ T Cell-Associated Gene Signature Correlates With Prognosis Risk and Immunotherapy Response in Patients With Lung Adenocarcinoma. Front Immunol. 2022; 13:806877. 10.3389/fimmu.2022.806877 35273597 PMC8902308

[13] Zhang S, Zhang W, Zhang J. 8-Gene signature related to CD8^+^ T cell infiltration by integrating single-cell and bulk RNA-sequencing in head and neck squamous cell carcinoma. Front Genet. 2022; 13:938611. 10.3389/fgene.2022.938611 35938006 PMC9355512

[14] Sun Y, Wu L, Zhong Y, Zhou K, Hou Y, Wang Z, Zhang Z, Xie J, Wang C, Chen D, Huang Y, Wei X, Shi Y, et al. Single-cell landscape of the ecosystem in early-relapse hepatocellular carcinoma. Cell. 2021; 184:404–21.e16. 10.1016/j.cell.2020.11.041 33357445

[15] Becht E, McInnes L, Healy J, Dutertre CA, Kwok IWH, Ng LG, Ginhoux F, Newell EW. Dimensionality reduction for visualizing single-cell data using UMAP. Nat Biotechnol. 2018. [Epub ahead of print]. 10.1038/nbt.4314 30531897

[16] Aran D, Looney AP, Liu L, Wu E, Fong V, Hsu A, Chak S, Naikawadi RP, Wolters PJ, Abate AR, Butte AJ, Bhattacharya M. Reference-based analysis of lung single-cell sequencing reveals a transitional profibrotic macrophage. Nat Immunol. 2019; 20:163–72. 10.1038/s41590-018-0276-y 30643263 PMC6340744

[17] Liu Z, Liu L, Weng S, Guo C, Dang Q, Xu H, Wang L, Lu T, Zhang Y, Sun Z, Han X. Machine learning-based integration develops an immune-derived lncRNA signature for improving outcomes in colorectal cancer. Nat Commun. 2022; 13:816. 10.1038/s41467-022-28421-6 35145098 PMC8831564

[18] Liu Z, Guo C, Dang Q, Wang L, Liu L, Weng S, Xu H, Lu T, Sun Z, Han X. Integrative analysis from multi-center studies identities a consensus machine learning-derived lncRNA signature for stage II/III colorectal cancer. EBioMedicine. 2022; 75:103750. 10.1016/j.ebiom.2021.103750 34922323 PMC8686027

[19] Zhang H, Zhang N, Wu W, Zhou R, Li S, Wang Z, Dai Z, Zhang L, Liu Z, Zhang J, Luo P, Liu Z, Cheng Q. Machine learning-based tumor-infiltrating immune cell-associated lncRNAs for predicting prognosis and immunotherapy response in patients with glioblastoma. Brief Bioinform. 2022; 23:bbac386. 10.1093/bib/bbac386 36136350

[20] Zhao B, Pei L. A macrophage related signature for predicting prognosis and drug sensitivity in ovarian cancer based on integrative machine learning. BMC Med Genomics. 2023; 16:230. 10.1186/s12920-023-01671-z 37784081 PMC10544447

[21] Li Z, Guo M, Lin W, Huang P. Machine Learning-Based Integration Develops a Macrophage-Related Index for Predicting Prognosis and Immunotherapy Response in Lung Adenocarcinoma. Arch Med Res. 2023; 54:102897. 10.1016/j.arcmed.2023.102897 37865004

[22] Thorsson V, Gibbs DL, Brown SD, Wolf D, Bortone DS, Ou Yang TH, Porta-Pardo E, Gao GF, Plaisier CL, Eddy JA, Ziv E, Culhane AC, Paull EO, et al, and Cancer Genome Atlas Research Network. The Immune Landscape of Cancer. Immunity. 2018; 48:812–30.e14. 10.1016/j.immuni.2018.03.023 29628290 PMC5982584

[23] Fu J, Li K, Zhang W, Wan C, Zhang J, Jiang P, Liu XS. Large-scale public data reuse to model immunotherapy response and resistance. Genome Med. 2020; 12:21. 10.1186/s13073-020-0721-z 32102694 PMC7045518

[24] Jiang P, Gu S, Pan D, Fu J, Sahu A, Hu X, Li Z, Traugh N, Bu X, Li B, Liu J, Freeman GJ, Brown MA, et al. Signatures of T cell dysfunction and exclusion predict cancer immunotherapy response. Nat Med. 2018; 24:1550–8. 10.1038/s41591-018-0136-1 30127393 PMC6487502

[25] Charoentong P, Finotello F, Angelova M, Mayer C, Efremova M, Rieder D, Hackl H, Trajanoski Z. Pan-cancer Immunogenomic Analyses Reveal Genotype-Immunophenotype Relationships and Predictors of Response to Checkpoint Blockade. Cell Rep. 2017; 18:248–62. 10.1016/j.celrep.2016.12.019 28052254

[26] Wang JY, Lu AQ, Chen LJ. LncRNAs in ovarian cancer. Clin Chim Acta. 2019; 490:17–27. 10.1016/j.cca.2018.12.013 30553863

[27] Bejarano L, Jordāo MJC, Joyce JA. Therapeutic Targeting of the Tumor Microenvironment. Cancer Discov. 2021; 11:933–59. 10.1158/2159-8290.CD-20-1808 33811125

[28] Dey P, Kimmelman AC, DePinho RA. Metabolic Codependencies in the Tumor Microenvironment. Cancer Discov. 2021; 11:1067–81. 10.1158/2159-8290.CD-20-1211 33504580 PMC8102306

[29] Jhunjhunwala S, Hammer C, Delamarre L. Antigen presentation in cancer: insights into tumour immunogenicity and immune evasion. Nat Rev Cancer. 2021; 21:298–312. 10.1038/s41568-021-00339-z 33750922

[30] Pan X, Bi F. A Potential Immune-Related Long Non-coding RNA Prognostic Signature for Ovarian Cancer. Front Genet. 2021; 12:694009. 10.3389/fgene.2021.694009 34367253 PMC8335165

[31] Liu L, Cai L, Liu C, Yu S, Li B, Pan L, Zhao J, Zhao Y, Li W, Yan X. Construction and Validation of a Novel Glycometabolism-Related Gene Signature Predicting Survival in Patients With Ovarian Cancer. Front Genet. 2020; 11:585259. 10.3389/fgene.2020.585259 33281878 PMC7689371

[32] Zhang J, Yang L, Xiang X, Li Z, Qu K, Li K. A panel of three oxidative stress-related genes predicts overall survival in ovarian cancer patients received platinum-based chemotherapy. Aging (Albany NY). 2018; 10:1366–79. 10.18632/aging.101473 29910195 PMC6046245

[33] Bi J, Bi F, Pan X, Yang Q. Establishment of a novel glycolysis-related prognostic gene signature for ovarian cancer and its relationships with immune infiltration of the tumor microenvironment. J Transl Med. 2021; 19:382. 10.1186/s12967-021-03057-0 34496868 PMC8425093

[34] Cheng Q, Li L, Yu M. Construction and validation of a transcription factors-based prognostic signature for ovarian cancer. J Ovarian Res. 2022; 15:29. 10.1186/s13048-021-00938-2 35227285 PMC8886838

[35] Wang H, Cheng Q, Chang K, Bao L, Yi X. Integrated Analysis of Ferroptosis-Related Biomarker Signatures to Improve the Diagnosis and Prognosis Prediction of Ovarian Cancer. Front Cell Dev Biol. 2022; 9:807862. 10.3389/fcell.2021.807862 35071242 PMC8766510

[36] Liang L, Li J, Yu J, Liu J, Xiu L, Zeng J, Wang T, Li N, Wu L. Establishment and validation of a novel invasion-related gene signature for predicting the prognosis of ovarian cancer. Cancer Cell Int. 2022; 22:118. 10.1186/s12935-022-02502-4 35292033 PMC8922755

[37] Yoshihara K, Shahmoradgoli M, Martínez E, Vegesna R, Kim H, Torres-Garcia W, Treviño V, Shen H, Laird PW, Levine DA, Carter SL, Getz G, Stemke-Hale K, et al. Inferring tumour purity and stromal and immune cell admixture from expression data. Nat Commun. 2013; 4:2612. 10.1038/ncomms3612 24113773 PMC3826632

[38] Lin W, Chen Y, Wu B, Chen Y, Li Z. Identification of the pyroptosis-related prognostic gene signature and the associated regulation axis in lung adenocarcinoma. Cell Death Discov. 2021; 7:161. 10.1038/s41420-021-00557-2 34226539 PMC8257680

[39] Doki Y, Ajani JA, Kato K, Xu J, Wyrwicz L, Motoyama S, Ogata T, Kawakami H, Hsu CH, Adenis A, El Hajbi F, Di Bartolomeo M, Braghiroli MI, et al, and CheckMate 648 Trial Investigators. Nivolumab Combination Therapy in Advanced Esophageal Squamous-Cell Carcinoma. N Engl J Med. 2022; 386:449–62. 10.1056/NEJMoa2111380 35108470

[40] Janjigian YY, Shitara K, Moehler M, Garrido M, Salman P, Shen L, Wyrwicz L, Yamaguchi K, Skoczylas T, Campos Bragagnoli A, Liu T, Schenker M, Yanez P, et al. First-line nivolumab plus chemotherapy versus chemotherapy alone for advanced gastric, gastro-oesophageal junction, and oesophageal adenocarcinoma (CheckMate 649): a randomised, open-label, phase 3 trial. Lancet. 2021; 398:27–40. 10.1016/S0140-6736(21)00797-2 34102137 PMC8436782

[41] Yang C, Xia BR, Zhang ZC, Zhang YJ, Lou G, Jin WL. Immunotherapy for Ovarian Cancer: Adjuvant, Combination, and Neoadjuvant. Front Immunol. 2020; 11:577869. 10.3389/fimmu.2020.577869 33123161 PMC7572849

[42] Samstein RM, Lee CH, Shoushtari AN, Hellmann MD, Shen R, Janjigian YY, Barron DA, Zehir A, Jordan EJ, Omuro A, Kaley TJ, Kendall SM, Motzer RJ, et al. Tumor mutational load predicts survival after immunotherapy across multiple cancer types. Nat Genet. 2019; 51:202–6. 10.1038/s41588-018-0312-8 30643254 PMC6365097

[43] Tian W, Lei N, Zhou J, Chen M, Guo R, Qin B, Li Y, Chang L. Extracellular vesicles in ovarian cancer chemoresistance, metastasis, and immune evasion. Cell Death Dis. 2022; 13:64. 10.1038/s41419-022-04510-8 35042862 PMC8766448

