# Supplementary material for: Machine learning constructs a T cell-related signature for predicting prognosis and drug sensitivity in ovarian cancer
Source: Aging (Albany NY). 2024 Feb 9;16(4):3332–49. doi: 10.18632/aging.205536 (PMC10929824; doi:10.18632/aging.205536)
Supplement: Supplementary Figures [file aging-16-205536-s001.pdf]

SUPPLEMENTARY FIGURES

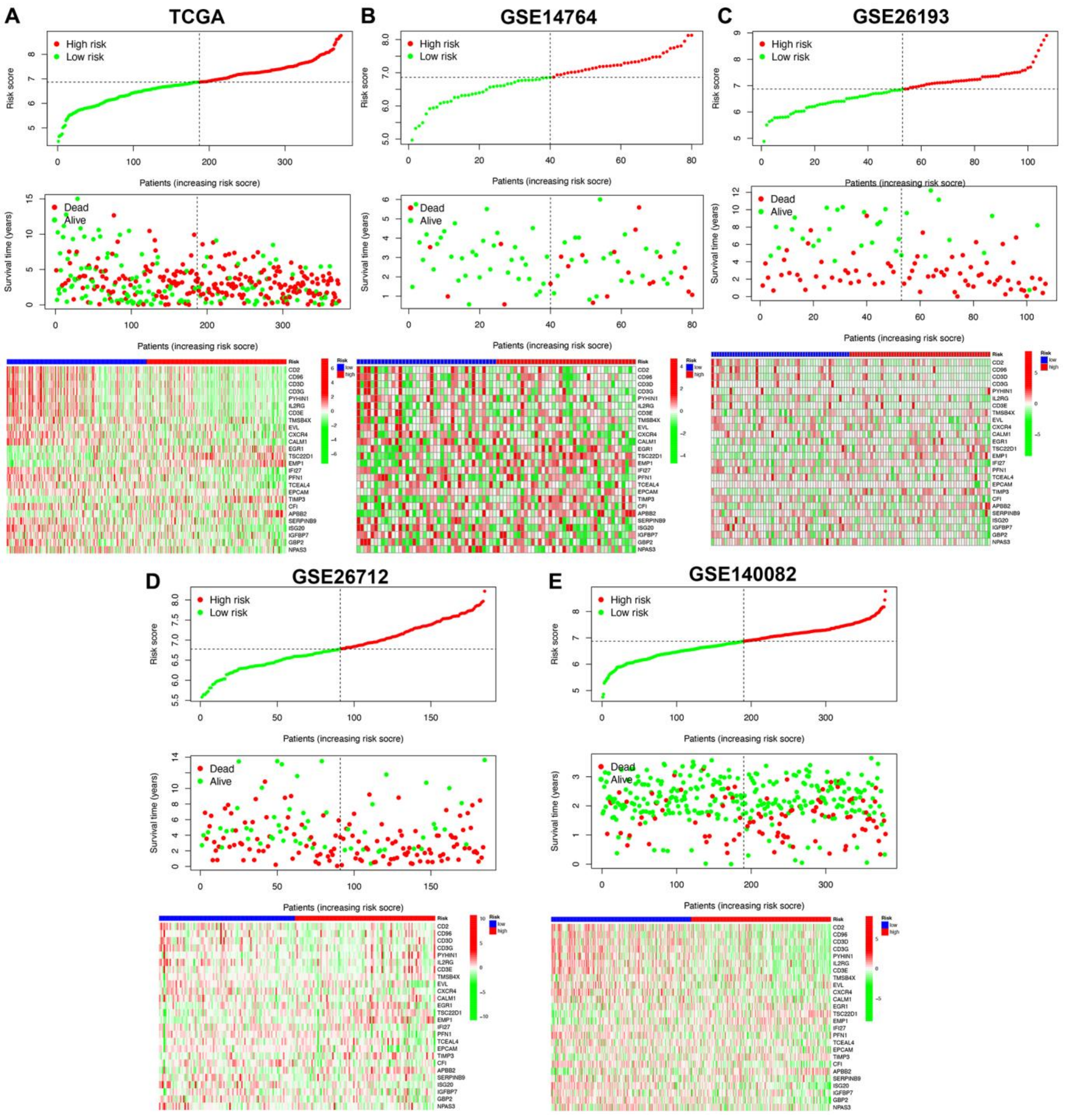

Supplementary Figure 1. Prognostic T cell-related signature (TRS) developed with integrative machine learning analysis. The risk score, survival status, and gene expression of TRS in TCGA (**A**), GSE14764 (**B**), GSE26193 (**C**), GSE26712 (**D**) and GSE140082 (**E**) cohort.

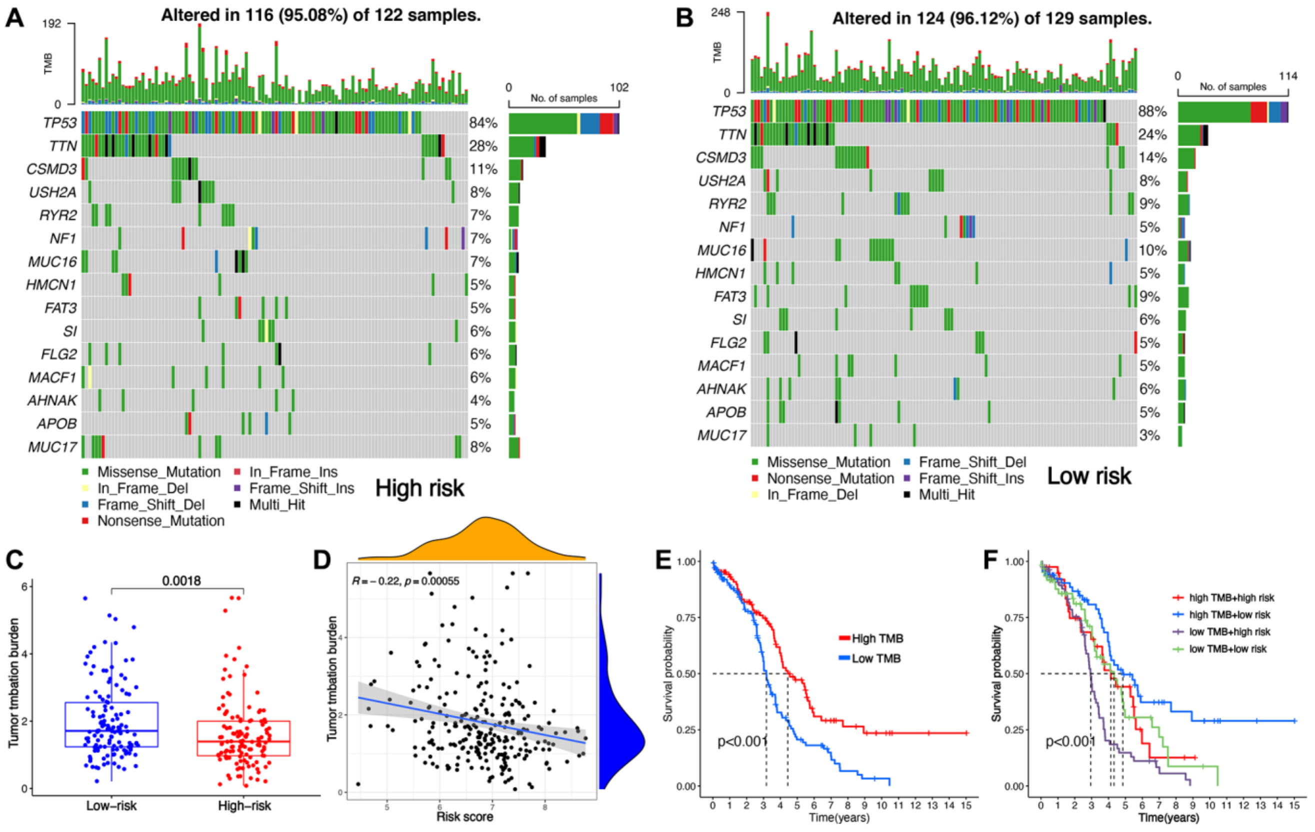

Supplementary Figure 2. Dissection of T cell-related signature (TRS)-based genetic mutation. (**A**, **B**) Genetic landscape in different risk score group of ovarian cancer. (**C**, **D**) The correlation between tumor mutational burden and risk score in ovarian cancer. (**E**, **F**) The overall survival curve in ovarian cancer patients with different tumor mutational burden and risk score.

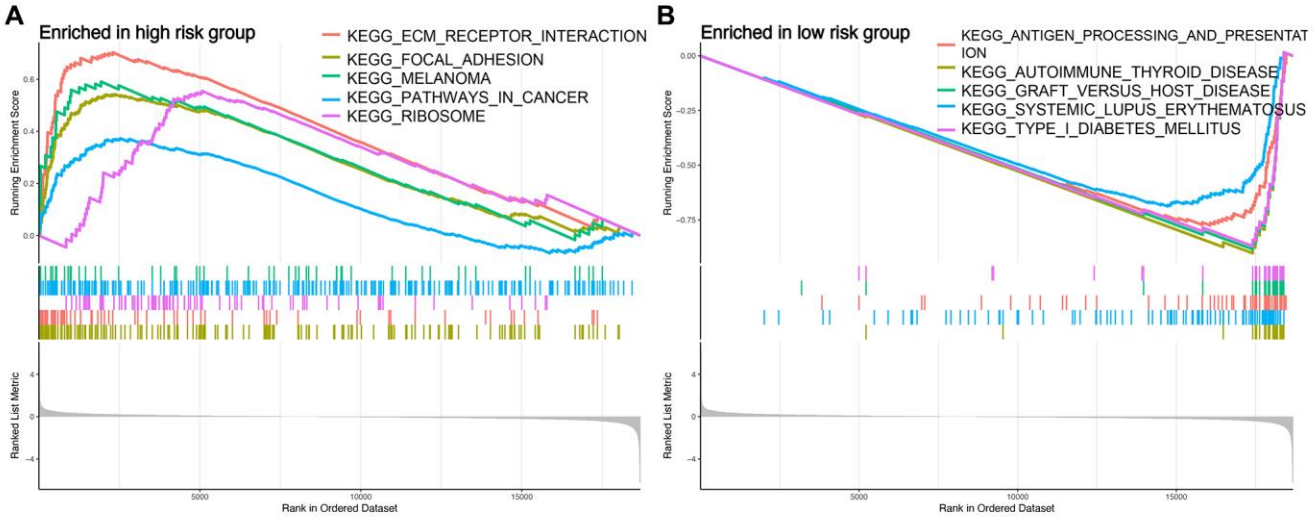

Supplementary Figure 3. T cell-related signature (TRS)-based GSEA analysis. The KEGG pathways associated enriched items in high (**A**) and low (**B**) risk score group.

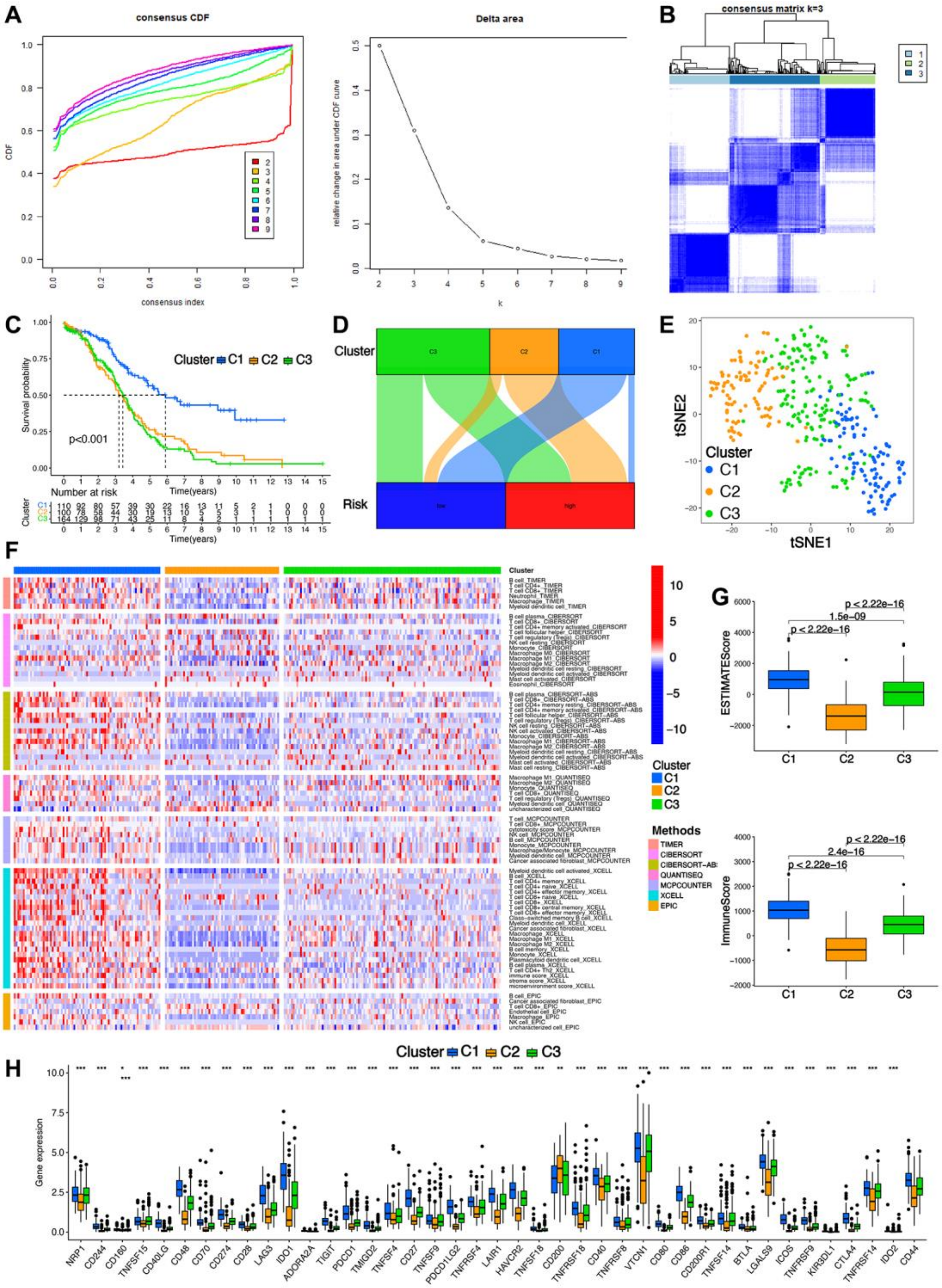

Supplementary Figure 4. Unsupervised clustering in different risk score group of ovarian cancer. (**A**, **B**) The consensus CDF, delta area and heatmap in unsupervised clustering analysis. (**C**) Survival curve of different clusters of ovarian cancer patients. (**D**) tSNE analysis demonstrated significant differences of gene profile of three clusters. (**E**) The correlation between risk score and cluster. The level of most of immune cells (**F**), tumor microenvironment score (**G**), and immune checkpoints (**H**) in different clusters of ovarian cancer patients.
