# Supplementary material for: Machine learning constructs a T cell-related signature for predicting prognosis and drug sensitivity in ovarian cancer
Source: Aging (Albany NY). 2024 Feb 9;16(4):3332–49. doi: 10.18632/aging.205536 (PMC10929824; doi:10.18632/aging.205536)
Supplement: Supplementary Tables 2 and 3 [file aging-16-205536-s003.pdf]

SUPPLEMENTARY TABLES

Supplementary Table 2. Univariate cox analysis identified 26 potential prognostic T cell-related markers.

Id	HR	HR.95L	HR.95H	*p*-value
CD2	0.865360154	0.773310799	0.968366402	0.011730077
CD96	0.81848001	0.682359338	0.981754759	0.030898357
CD3D	0.855767714	0.761840568	0.961275117	0.00864525
CD3G	0.745756741	0.622389088	0.893577871	0.001475151
PYHIN1	0.790027811	0.624398349	0.999592558	0.049604496
IL2RG	0.880170893	0.789195015	0.981634178	0.021850169
CD3E	0.847826179	0.731858414	0.982169796	0.027828459
TMSB4X	0.830523565	0.700504437	0.984675264	0.03253733
EVL	0.773898077	0.609409003	0.982785338	0.035518711
CXCR4	0.84538217	0.739302911	0.966682266	0.014076995
CALM1	0.772908781	0.634598006	0.941364419	0.010448589
EGR1	1.123864428	1.004779747	1.257062811	0.041012343
TSC22D1	1.245954265	1.024908258	1.514674137	0.027322858
EMP1	1.352393712	1.138702904	1.606186079	0.000581297
IFI27	0.913242335	0.846203341	0.985592377	0.019646104
PFN1	0.728993077	0.55948889	0.949850686	0.019231486
TCEAL4	0.788075267	0.635526762	0.977240714	0.030029456
EPCAM	0.833101003	0.716145292	0.969157081	0.017987523
TIMP3	1.160659569	1.043456987	1.291026512	0.006084275
CFI	0.848911225	0.738890634	0.975313848	0.020727341
APBB2	1.246524579	1.007132053	1.542820052	0.042838551
SERPINB9	0.806424564	0.65568386	0.991820322	0.041574256
ISG20	0.755275505	0.644744872	0.88475475	0.000507656
IGFBP7	0.802360401	0.684443615	0.940592035	0.00662439
GBP2	0.863193103	0.753796435	0.988466247	0.033358176
NPAS3	0.853256562	0.736786745	0.988137702	0.034062684

Supplementary Table 3. Other models had been established for ovarian cancer.

Signature	Title	PMID
An	Development of a Novel Autophagy-related Prognostic Signature for Serous Ovarian Cancer	30410611
Any	The Comprehensive Analysis of Interferon-Related Prognostic Signature with regard to Immune Features in Ovarian Cancer.	35769811
Bi	Establishment of a novel glycolysis-related prognostic gene signature for ovarian cancer and its relationships with immune infiltration of the tumor microenvironment	34496868
Chaofan	Establishment and validation of an RNA binding protein-associated prognostic model for ovarian cancer	33550985
Chen	Integrating cell cycle score for precise risk stratification in ovarian cancer	36061171
Cheng	Construction and validation of a transcription factors-based prognostic signature for ovarian cancer.	35227285
Fan	A newly defined risk signature, consisting of three m6A RNA methylation regulators, predicts the prognosis of ovarian cancer	32950970
Fei	Construction autophagy-related prognostic risk signature to facilitate survival prediction, individual treatment and biomarker excavation of epithelial ovarian cancer patients.	33676525
Hu	Identification of a five-gene signature of the RGS gene family with prognostic value in ovarian cancer.	33845140

Huan	Integrated Analysis of Ferroptosis-Related Biomarker Signatures to Improve the Diagnosis and Prognosis Prediction of Ovarian Cancer	35071242
Huo	Identification of a Prognostic Signature for Ovarian Cancer Based on the Microenvironment Genes	34054929
Jin	A panel of three oxidative stress-related genes predicts overall survival in ovarian cancer patients received platinum-based chemotherapy	29910195
JinC	A 2-Protein Signature Predicting Clinical Outcome in High-Grade Serous Ovarian Cancer	28976449
Jinwei	Identification and verification of a ten-gene signature predicting overall survival for ovarian cancer	32805252
Khadirnaikar	Development and validation of an immune prognostic signature for ovarian carcinoma	32794637
Lei	Identification of an energy metabolism-related gene signature in ovarian cancer prognosis	32186777
Leilei	Establishment and validation of a novel invasion-related gene signature for predicting the prognosis of ovarian cancer.	35292033
Liang	A Novel Glycosyltransferase-Related Gene Signature for Overall Survival Prediction in Patients with Ovarian Cancer	34992448
Lin	A methylation-driven genes prognostic signature and the immune microenvironment in epithelial ovarian cancer.	35783253
Liu	Construction and validation of a novel aging-related gene signature and prognostic nomogram for predicting the overall survival in ovarian cancer.	34825509
Lixiao	Construction and Validation of a Novel Glycometabolism-Related Gene Signature Predicting Survival in Patients With Ovarian Cancer.	33281878
Nie	Prognostic signature of ovarian cancer based on 14 tumor microenvironment-related genes	34260536
Pan	A Potential Immune-Related Long Non-coding RNA Prognostic Signature for Ovarian Cancer	34367253
Pan	A Novel Six-Gene Signature for Prognosis Prediction in Ovarian Cancer	33193589
Qiu	A Liquid-Liquid Phase Separation-Related Gene Signature as Prognostic Biomarker for Epithelial Ovarian Cancer	34168991
